# Supplementary material for: Can quantum computers do nothing?
Source: npj Quantum Inf. 2024 Nov 26;10(1):124. doi: 10.1038/s41534-024-00918-6 (PMC11599043; doi:10.1038/s41534-024-00918-6)
Supplement: Supplementary file 1 — Revised Supplementary Material [file 41534_2024_918_MOESM1_ESM.pdf]

Supplementary Material: Can Quantum Computers Do Nothing?

Alexander Nico-Katz,[1,2,*] Nathan Keenan,[1,2,3,†] and John Goold[1,2,4,‡]

[1]School of Physics, Trinity College Dublin, Dublin 2, Ireland

[2]Trinity Quantum Alliance, Unit 16, Trinity Technology and Enterprise Centre, Pearse Street, D02 YN67, Dublin 2, Ireland

[3]IBM Quantum, IBM Research Europe - Dublin, IBM Technology Campus, Dublin 15, Ireland

[4]Algorithmiq Ltd, Kanavakatu 3C 00160, Helsinki, Finland

(Dated: July 2, 2024)

I. ADDITIONAL RESULTS

Here we present additional results in Fig. 1 for the statistics of $\Delta\chi^{SP}$ and $\Delta\chi^{SR}$ (see main text) at different shot counts N_S. Dashed blue and dotted orange lines indicate the $K = 4$ box-filtration fences according to the outlier filtering discussed in the main text for $\Delta\chi^{SP}$ and $\Delta\chi^{SR}$ respectively. In all cases we identify a bump in $P(\Delta\chi^{SP})$ at $\Delta\chi^{SP} \gtrsim 0.12$, for the nearest-neighbour complementary set of qubits P *only*. As discussed in the main text, this bump corresponds to 'bad qubits' which leak considerable information (over 10% of a classical bit) into their surroundings. The fact that this bump is only visible in the nearest-neighbour complementary set data P, and that it doesn't change position with changing shot count, suggests that it is entirely independent of shot noise or other sources of error and is in fact a direct quantification of idle information loss.

We also note that, as the shout count is doubled between the panels Fig. 1**(a)-(d)**, the centre of the main Gaussian distribution decreases; indicating that much of the signal is dominated by shot noise. We evaluate the mean values $\overline{\Delta\chi}$ and their respective standard errors in the mean, both of which are shown in the inset boxes in each panel. The results of one-tailed Welch's t-tests are summarized in the main text, indicating that the statistics for $\overline{\Delta\chi^{SP}}$ and $\overline{\Delta\chi^{SR}}$ are different to a high degree of statistical significance: with z-values in excess of $z = 5$ for all the results shown in Fig. 1. This indicates a positive detection of idle information loss at all shot counts investigated; even if the absolute value of this idle information loss is very small.

Finally, we note that at the higher shout counts $N_S = 32000$ and $N_S = 64000$ shown in Fig. 1**(c)** and Fig. 1**(d)** respectively, there is a finite probability of locating some results outside of the filter boundary but well below the threshold of $\Delta\chi \gtrsim 0.12$ for unambiguous 'bad qubits'. This corresponds to qubits which are leaking enough information to be visible above the background of shot noise but which may not have failed completely.

* nicokata@tcd.ie

† nakeenan@tcd.ie

‡ gooldj@tcd.ie

II. DETAILS OF STATE TOMOGRAPHY

State tomography in IBM devices is carried out by measuring all M qubits in the output register $j_1, j_2, \cdots, j_M$, where j_r indexes the physical location of the r-th output qubit, simultaneously in the three Pauli bases. Taking the standard Pauli matrices, $\sigma^0 = \mathbb{I}$, $\sigma^1 = X$, $\sigma^2 = Y$, $\sigma^3 = Z$, the statistics of each Pauli string $P_b = \sigma_{j_1}^{b_1}\sigma_{j_2}^{b_2}\cdots\sigma_{j_M}^{b_M}$ with M non-identity elements ($b_r \neq 0$ such that there are 3^M of this family of string in total) is determined by these measurements. The result of each measurement is a bitstring, and the result of a large number N_S of measurements - called 'shots' - is a dictionary D_b of bitstrings. Taking N_S sufficiently large ensures that the sample statistics of measuring a specific bitstring given the Pauli string P_b are close to the population statistics. These statistics for the reduced space of Pauli strings can then be aggregated into marginal values which yield statistics for the full space of 4^M Pauli strings. This is done by simply aggregating shots from different Pauli strings together wherever they coincide everywhere except where identities occur in the desired marginal.

Shot Number N_S	Complementary Set	Samples
4000	P	609
	R	600
8000	P	507
	R	480
16000	P	324
	R	288
32000	P	252
	R	204
64000	P	157
	R	157

TABLE I. **Total sample counts for all experimental implementations** Sample counts, before filtering, for each shot count and type of tomographic complementary set: nearest-neigbour P and random (excluding nearest-neighbour) R. Each sample corresponds to a single realization of steps 1-7 of the protocol discussed in the main text.

As a concrete example, consider an output register of three qubits, and lets say we are interested in the value of $\langle XIY\rangle$. This is given by the statistics of $XXY = P_1$, $XYY = P_2$, and $XZY = P_3$. For each of these P_b statistics, there is a corresponding dictionary of results

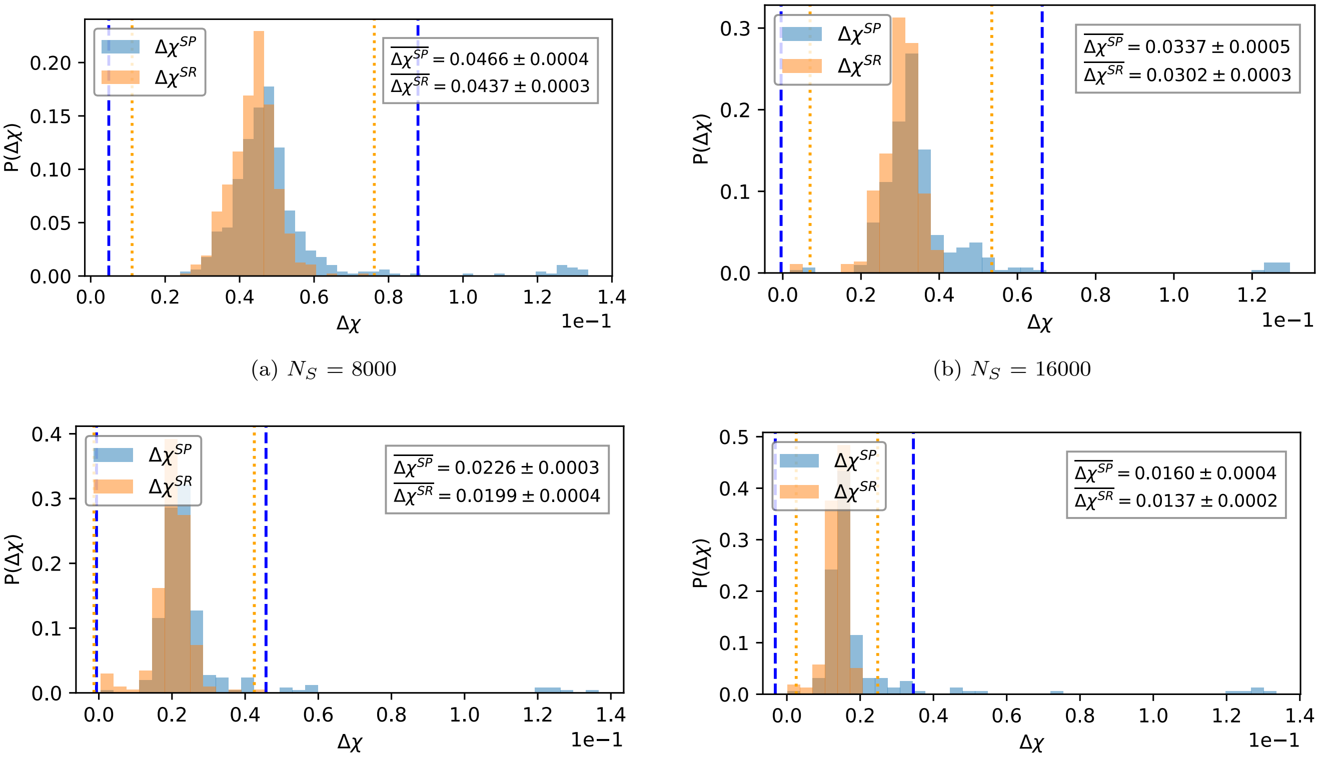

(a) $N_S = 8000$

(b) $N_S = 16000$

(c) $N_S = 32000$

(d) $N_S = 64000$

FIG. 1. **Experimental sample statistics for** $\Delta\chi$ Statistics of $\Delta\chi^{SP}$ and $\Delta\chi^{SR}$ for nearest-neighbour P (blue) and random R (orange) complementary sets of qubits respectively. Different shot counts N_S shown in **(a)** $N_S = 8000$, **(b)** $N_S = 16000$, **(c)** $N_S = 32000$, **(d)** $N_S = 64000$. The ranges determined by a box-fitting tolerance of $K = 4$ (see main text) are shown as blue dashed lines for $\Delta\chi^{SP}$, and orange dotted lines for $\Delta\chi^{SR}$. Inset shows means and standard errors for the non-outlier region of each distribution. A noticable bump of outliers at larger values of *only* $\Delta\chi^{SP}$ is visible in all cases. Statistics are comprised of 2369 total samples, taken across four months (Dec 2023 - Mar 2024) from all the Falcon 5.11 series of devices.

$D_b = \{B_b^s\}$ where the B_b^s are bitstrings x which are simply the results of any single shot (measurement) given b. The statistics of P_1 are given by calculation of the probability distribution $P_b(B_b = x) = P(B = x|b)$. An example dictionary for P_1 for $N_S = 10$ shots might be

$$D_1 = \{101, 101, 101, 111, 001, 101, 101, 001, 101, 100\} \quad (1)$$

with an associated probability distribution

$$P_1(B_1 = x) = \begin{cases} 0.6 & \text{if } x = 101 \\ 0.1 & \text{if } x = 111 \\ 0.2 & \text{if } x = 001 \\ 0.1 & \text{if } x = 100 \\ 0.0 & \text{otherwise} \end{cases} . \quad (2)$$

Now consider the following example dictionaries for P_2 and P_3:

$$D_2 = \{111, 101, 111, 111, 011, 111, 101, 011, 101, 101\} \quad (3)$$

$$D_3 = \{101, 111, 011, 111, 011, 110, 111, 001, 101, 110\}. \quad (4)$$

We then aggregate the dictionaries D_b into a single new dictionary $\widetilde{D}$ which describes the statistics of XIY by simply excluding the central bit of each bitstring and aggregating the dictionaries:

$$D_1 \to \{11, 11, 11, 11, 01, 11, 11, 01, 11, 10\} \quad (5)$$
$$D_2 \to \{11, 11, 11, 11, 01, 11, 11, 01, 11, 11\} \quad (6)$$
$$D_3 \to \{11, 11, 01, 11, 01, 10, 11, 01, 11, 10\} \quad (7)$$
$$\widetilde{D} = D_1 + D_2 + D_3 \quad (8)$$

where $\widetilde{D}$ contains 30 elements. The statistics $\widetilde{P}(\widetilde{B} = \widetilde{x})$ of $\widetilde{D}$ are then calculated as

$$\widetilde{P}(\widetilde{B} = \widetilde{x}) = \begin{cases} 0.6\dot{6} & \text{if } x = 11 \\ 0.2\dot{3} & \text{if } x = 01 \\ 0.10 & \text{if } x = 10 \\ 0.00 & \text{otherwise} \end{cases} . \quad (9)$$

We can now evaluate $\langle XIY \rangle$ explicitly by summing up contributions to the expectation value $1 \to 1$, $0 \to -1$ as

follows

$$\langle XIY \rangle = 0.6\dot{6}(-1\times-1)+0.2\dot{3}(1\times-1)+0.1(-1\times0) = 0.\dot{3} \quad (10)$$

which completes our example.

This may seem a laborious process, but it allows us to extrapolate $\mathcal{O}(4^M)$ elements of a given state's density matrix using only $\mathcal{O}(N_S 3^M)$ measurements. By decomposing the state's density matrix into a sum of 4^M Pauli strings (including identity elements),

$$\hat{\rho} = \frac{1}{\mathcal{Z}} \sum_b \langle P_b \rangle P_b \quad (11)$$

where $\mathcal{Z}$ is an appropriate normalization factor, we can readily reconstruct the quantum state of the output register using the dictionaries D_k. These dictionaries D_k are ultimately what IBM's quantum computers return to their users. Where we discuss shot count N_S in the main text, it simply refers the size of these returned dictionaries; where larger dictionaries more accurately yield the statistics of the actual population. The total number of samples of $\Delta\chi$ for both complementary qubit sets discussed in the main text (see main text) is shown in Table I for all shot counts N_S we consider.

III. APHYSICALITY AND MAXIMUM-LIKELIHOOD RECONSTRUCTION

As discussed in the main text, shot noise due to finite N_S can result in aphysical density matrices by introducing negative eigenvalues into their spectra. We correct for this using the maximum-likelihood reconstruction of the density matrix.

The tomographic process discussed in Section II yields a density matrix $\hat{\mu}$ with matrix elements μ_{ij} which is definitionally of trace unity and hermitian by inspection of Eq. (11). The eigenvalues μ_j of $\hat{\mu}$ can, however, be negative; and thus $\hat{\mu}$ is generally unphysical.

We follow and briefly summarize here the maximum-likelihood mixed state reconstruction algorithm given in the work of Smolin et al. [1]. First we invoke the existence of some density matrix $\hat{\rho} \in \mathcal{P}$ with matrix elements ρ_{ij} and eigenvalues ρ_j which minimizes the 2-norm,

$$\min_{\hat{\rho}\in\mathcal{P}} \parallel \hat{\rho} - \hat{\mu} \parallel_2^2 = \min_{\hat{\rho}\in\mathcal{P}} \sum_{ij} |\rho_{ij} - \mu_{ij}|^2 \quad (12)$$

where $\mathcal{P}$ is the space of physical density matrices (unit trace, positive semi-definite, hermitian matrices). We remark here that Eq. (12) is invariant under change of basis, and hence we choose to work in the eigenbasis of $\hat{\mu}$ with eigenvectors $|\mu_j\rangle$ such that,

$$\parallel \hat{\rho} - \hat{\mu} \parallel_2^2 = \sum_{ij} |\rho_{ij} - \mu_j \delta_{ij}|^2 \quad (13)$$

where δ_{ij} is the Kronecker delta. Clearly, Eq. (13) is minimized when $\hat{\rho}$ is also diagonal in this basis, i.e. the eigenvectors of $\hat{\rho}$ are also $|\mu_j\rangle$, as any non-zero off-diagonal terms ρ_{ij} for $i \neq j$ strictly increases the value of Eq. (13). This reduces the minimization procedure down from an $\mathcal{O}(\Omega^2)$ problem (where Ω is the total dimension of the system) to a minimization problem in the $\Omega - 1$ eigenvalues of $\hat{\rho}$,

$$\min_{\hat{\rho}\in\mathcal{P}} \parallel \hat{\rho} - \hat{\mu} \parallel_2^2 = \min_{\{\rho_j\}} \sum_j |\rho_j - \mu_j|^2 \quad (14)$$

subject to only two constraints: that $\rho_j \geq 0$, and that $\sum_j \rho_j = 1$. The reconstructed density matrix is finally given by

$$\hat{\rho} = \sum_j \rho_j |\mu_j\rangle\langle\mu_j|. \quad (15)$$

For the system sizes that we consider, the $\mathcal{O}(\Omega)$ minimization problem can be solved quickly by standard numerical minimization packages. This is the approach we use in the main text of this article. For larger problems, Smolin et al. provide a simple algorithm after reducing the complexity of the problem further by noting that the solution to Eq. (14) essentially involves finding a 'pivot' j' in the (ordered) μ_j wherein $\rho_j = 0$ for $j < j'$ and $\rho_j = \mu_j + c$ where c is a constant for $j \geq j'$. The use of this algorithm is unnecessary for the situations we consider in this article, and we refer the interested reader to [1] for more details.

[1] J. A. Smolin, J. M. Gambetta, and G. Smith, Efficient method for computing the maximum-likelihood quantum state from measurements with additive gaussian noise, Phys. Rev. Lett. **108**, 070502 (2012).
